# *In vivo* genome editing with a small Cas9 orthologue derived from *Campylobacter jejuni*

**DOI:** 10.1038/ncomms14500

**Published:** 2017-02-21

**Authors:** Eunji Kim, Taeyoung Koo, Sung Wook Park, Daesik Kim, Kyoungmi Kim, Hee-Yeon Cho, Dong Woo Song, Kyu Jun Lee, Min Hee Jung, Seokjoong Kim, Jin Hyoung Kim, Jeong Hun Kim, Jin-Soo Kim

**Affiliations:** 1 Center for Genome Engineering, Institute for Basic Science (IBS), Seoul 08826, Republic of Korea; 2 ToolGen, Byucksan Digital Valley 6-cha, 219 Gasan Digital 1-ro, Geumcheon-gu, Seoul 08501, Republic of Korea; 3 Department of Functional Genomics, University of Science and Technology, Daejeon 34113, Republic of Korea; 4 Department of Biomedical Sciences, Seoul National University College of Medicine, Seoul 03080, Republic of Korea; 5 FARB Laboratory, Biomedical Research Institute, Seoul National University Hospital, Seoul 03082, Republic of Korea; 6 Department of Chemistry, Seoul National University, Seoul 08826, Republic of Korea; 7 Department of Ophthalmology, Seoul National University College of Medicine, Seoul 03080, Republic of Korea

## Abstract

Several CRISPR-Cas9 orthologues have been used for genome editing. Here, we present the smallest Cas9 orthologue characterized to date, derived from *Campylobacter jejuni* (CjCas9), for efficient genome editing *in vivo*. After determining protospacer-adjacent motif (PAM) sequences and optimizing single-guide RNA (sgRNA) length, we package the CjCas9 gene, its sgRNA sequence, and a marker gene in an all-in-one adeno-associated virus (AAV) vector and produce the resulting virus at a high titer. CjCas9 is highly specific, cleaving only a limited number of sites in the human or mouse genome. CjCas9, delivered via AAV, induces targeted mutations at high frequencies in mouse muscle cells or retinal pigment epithelium (RPE) cells. Furthermore, CjCas9 targeted to the *Vegfa* or *Hif1a* gene in RPE cells reduces the size of laser-induced choroidal neovascularization, suggesting that *in vivo* genome editing with CjCas9 is a new option for the treatment of age-related macular degeneration.

## Abstract (web summary)

The amount of genetic material that can be packaged in AAV vectors used for genome editing is limited. Here the authors show that the smallest known Cas9 orthologue, cjCas9, can be packaged in a single AAV vector along with sgRNA and a marker gene, and demonstrate efficient gene editing in mice.

Genome editing has recently been democratized by the development of RNA-guided programmable nucleases repurposed from the type II clustered regularly interspaced short palindromic repeats (CRISPR)/CRISPR-associated (Cas) adaptive immune system against invading genetic elements in eubacteria and archaea^1^. Cas9, the single effector protein component in the system, is complexed with CRISPR RNA (crRNA) and trans-activating crRNA (tracrRNA) or with single-guide RNA (sgRNA) composed of essential portions of crRNA and tracrRNA to form a sequence-specific RNA-guided endonuclease (RGEN)^2^. A new RGEN with desired target specificity is readily prepared by replacing crRNA or sgRNA, which hybridizes with a target DNA sequence. Cas9 RGENs cleave chromosomal DNA in a targeted manner, enabling genetic modifications or genome editing in cells and whole organisms^3^,^4^,^5^,^6^,^7^,^8^.

Cas9 derived from *Streptococcus pyogenes* (SpCas9), the first Cas9 orthologue to enable targeted mutagenesis in human cells^3^,^5^,^6^,^9^, is still the most widely used among several Cas9 proteins available for genome editing. Owing to it large size (1,368 amino acids, 4.10 kbp; Fig. 1a), however, the SpCas9 gene and its sgRNA sequence cannot be packaged together into certain viral vectors such as adeno-associated virus (AAV)^10^ for efficient delivery into cells *in vivo*. Instead, the SpCas9 gene alone can be packaged into a single AAV vector *in vivo*^11^,^12^. Alternatively, SpCas9 can be split into two parts^13^,^14^, each can be packaged into two AAV vectors^15^,^16^. However, split SpCas9 is less active than the intact SpCas9 (refs 13, 14). Furthermore, co-delivery of two AAV vectors is less efficient than the delivery of a single AAV vector *in vivo*.

As an alternative to SpCas9, *Staphylococcus aureus* Cas9 (SaCas9) can be used for genome editing^17^. SaCas9 is smaller (1,053 amino acids, 3.16 kbp) than SpCas9 and can be packaged, together with its sgRNA, into an AAV vector. Still, the SaCas9 gene cannot be packaged with a florescent reporter gene or two sgRNAs, essential for targeted chromosomal deletions or other rearrangements, into a single AAV vector *in vivo*. Furthermore, protospacer-adjacent motif (PAM) sequences recognized by SaCas9, 5′-NNGRRT-3′, occur less frequently than those recognized by SpCas9, 5′-NGG-3′, limiting targetable sites. In this regard, smaller Cas9 orthologues with different PAM sequences are highly desired to expand *in vivo* genome editing. Here, we present *Campylobacter jejuni*-derived Cas9 (CjCas9) for efficient genome editing *in vitro* and *in vivo*. CjCas9 is composed of 984 amino-acid residues (2.95 kbp) (Supplementary Fig. 1), much smaller than SpCas9 or SaCas9 (Fig. 1a), but has never been shown to induce targeted mutagenesis in human or other eukaryotic cells or organisms. In this study, we characterized PAM sequences and optimized the sgRNA length to utilize CjCas9 for genome editing in mice.

## Results

### Determination of PAM sequences recognized by CjCas9

**Figure 1:**
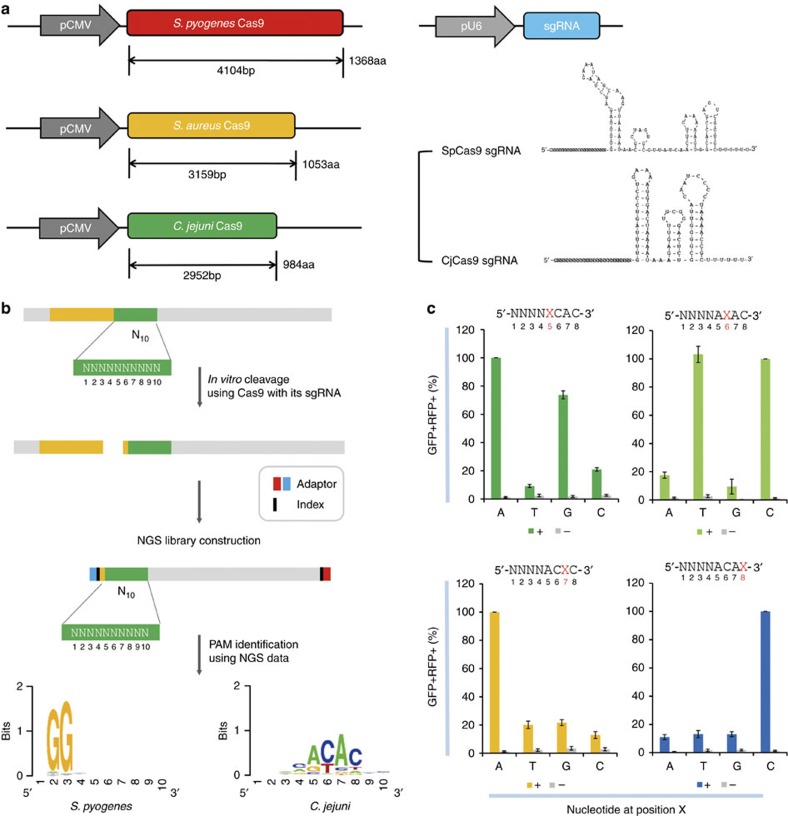
CjCas9 and its PAM specificity. (**a**) Cas9 orthologues and their sgRNAs. sgRNA structures were obtained using the mfold web server. See also Supplementary Fig. 1. (**b**) Schematic showing a PAM assay for characterization of PAM sequences recognized by Cas9. PCR amplicons containing a Cas9 target sequence (yellow) followed by randomized 10-bp (N_10_) sequences (green) were cleaved by CjCas9 *in vitro*. The sequence logo showing the PAM specificity was obtained using deep-sequencing data. (**c**) PAM specificity in cells. Surrogate reporters encoding GFP and RFP and containing a CjCas9 target sequence with degenerate PAMs were transfected with CjCas9 and its sgRNA plasmid into HEK 293 cells. The percentage of cells that express both GFP and RFP was normalized with that obtained using the optimal reporter containing the 5'-NNNNACAC-3' PAM sequence. (−) represents a mock control. Error bars indicate s.e.m. (*n*=3).

First, we determined the PAM sequences recognized by CjCas9 *in vitro* (Fig. 1b). A PCR amplicon library containing a CjCas9 target sequence followed by randomized 10-base pair (bp) sequences was cleaved by CjCas9 (and SpCas9 as a control) and a sgRNA specific to the target sequence. Cleaved duplexes were subjected to deep sequencing to identify PAM sequences recognized by CjCas9. This assay revealed that CjCas9 recognized 5′-NNNNACAC-3′ or 5′-NNNNRYAC-3′ (where R and Y stands for purines and pyrimidines, respectively) as PAMs *in vitro*. Using a different *in vitro* assay, Fonfara *et al*.^18^ reported that the optimal PAM for CjCas9 was 5′-NNNNACA-3′.

To confirm the PAM specificity in human cells, we performed cell-based reporter assays by co-transfecting plasmids encoding CjCas9 and its sgRNA and reporter plasmids containing the target site with variable PAM sequences between RFP and GFP sequences^19^ (Fig. 1c). Because the RFP sequence is fused to the GFP sequence out of frame in these reporter plasmids, cells express RFP but not GFP (RFP+ GFP−) in the absence of CjCas9. When CjCas9 cleaves the target site and induces small insertions or deletions (indels), the GFP sequence can be fused to the RFP sequence in frame. These reporter assays showed that CjCas9 cleaved target sites containing 5′-NNNNRYAC-3′ PAM sequences in HEK 293 cells. Importantly, the cytosine nucleotide at the 3′-end was essential.

### Optimization of CjCas9 sgRNA length

**Figure 2:**
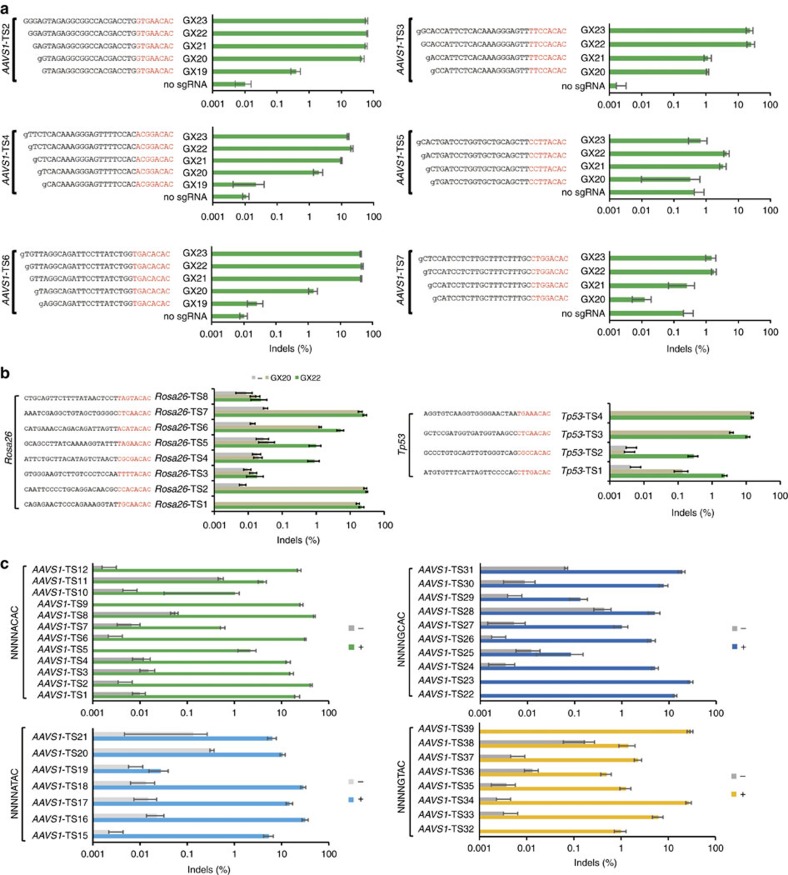
Optimization of sgRNA length for CjCas9. (**a**) sgRNAs with variable lengths (19 to 23 nucleotide complementary with a target DNA sequence) were designed and transfected with CjCas9 plasmid into human HEK 293 cells. Genomic DNA was isolated 48 h after transfection. Indel frequencies were analysed by targeted deep sequencing. The first guanine nucleotide at the 5' end that does not match the target sequence is shown in lower case. PAM motifs are shown in red. Error bars indicate s.e.m. (*n*=3). (**b**) Mutation frequencies at target sites in the mouse genome. *Rosa26* and *Tp53*-specific gX_22_ guide RNAs were designed and transfected into mouse NIH 3T3 cells together with CjCas9 plasmid. Genome editing efficiencies were examined by deep sequencing using genome DNA isolated from cells after 48 h of transfection. PAM motifs are shown in red. Error bars indicate s.e.m. (*n*=3). See also Supplementary Fig. 2. (**c**) CjCas9-mediated genome editing at the human *AAVS1* locus with different PAM sequences. sgRNAs targeting sites with a 5'-NNNNACAC-3' PAM (green; 12 sgRNAs), 5′-NNNNATAC-3′ PAM (light blue; 7 sgRNAs), 5′-NNNNGCAC-3′ PAM (dark blue; 10 sgRNAs), and 5′-NNNNGTAC-3′ PAM (yellow; 8 sgRNAs) were designed and their activities examined in HEK293 cells with deep sequencing. Error bars indicate s.e.m. (*n*=3).

We next optimized the sgRNA length for CjCas9-mediated genome editing in human and mouse cells (Fig. 2; Supplementary Fig. 2; Supplementary Table 1). We co-transfected CjCas9 and a series of sgRNAs with variable lengths into HEK 293 cells or NIH 3T3 cells and measured indel frequencies using targeted amplicon sequencing. The first nucleotide in the sgRNAs was fixed to an extra guanine (G) because sgRNAs are transcribed under the control of the U6 promoter, which requires a guanine at the 5′ end. Unlike SpCas9, which is most active with GX_20_ sgRNAs that hybridize with a 20-nucleotide target DNA sequence upstream of a PAM, CjCas9 was most active with GX_22_ sgRNAs that hybridize with a 22 nucleotides target sequence. GX_20_ and GX_19_ sgRNAs failed to induce indels at 8 out of 19 sites and 3 out of 4 sites, respectively in human or mouse cells.

We tested GX_22_ sgRNAs at 12 sites containing the optimal 5′-NNNNACAC-3′ PAM in human cells. All of these sgRNAs co-transfected with CjCas9 were able to induce indels at frequencies that ranged from 1.0 to 64% (21±5%, on average). CjCas9 also induced indels at sites with 5′-NNNNGCAC-3′ PAMs, 5′-NNNNGTAC-3′ PAMs, and 5′-NNNNATAC-3′ PAMs albeit less efficiently (10±3%, 10±4%, and 16±5%, respectively).

### Genome-wide target specificities of CjCas9

**Figure 3:**
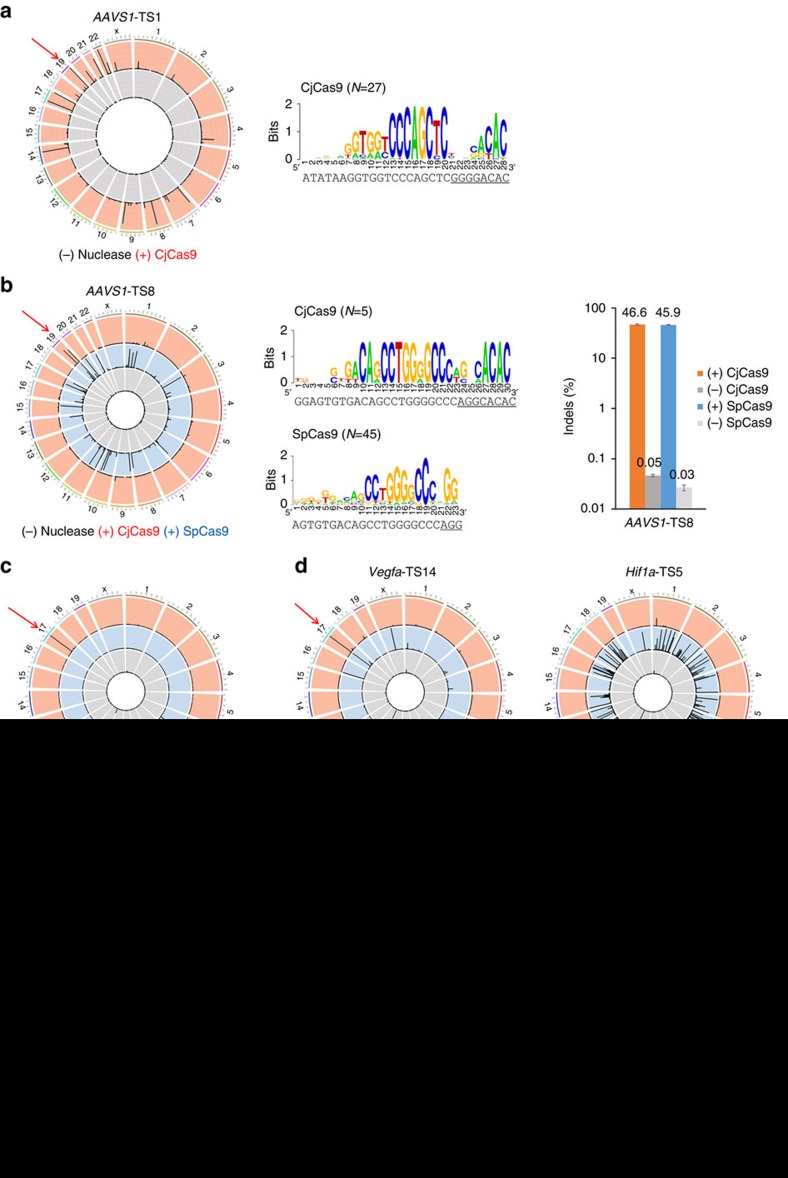
Genome-wide target specificities of CjCas9 nucleases examined using Digenome-seq. Human or mouse genomic DNA isolated from HeLa cells or NIH 3T3 cells (grey), respectively, was digested *in vitro* by Cas9 and its sgRNA targeted to the human *AAVS1* locus (**a**,**b**,**f**) and the mouse *Rosa26* (**c**), *Vegfa* (**c**,**d**) and *Hif1a* loci (**d**) and subjected to whole-genome sequencing. Circos plots show genome-wide DNA cleavage scores across the human or mouse genome. Red arrows indicate on-target sites. *N* indicates the number of *in vitro* cleavage sites identified by Digenome-seq. (**a**,**b**) Sequence logos were obtained by comparing DNA sequences at *in vitro* cleavage sites with each other. (**b**) Indel frequencies at the *AAVS1*-TS8 site measured using targeted deep sequencing. CjCas9 (orange) or SpCas9 (blue) targeted to the *AAVS1*-TS8 site was transfected into human HEK293 cells. Error bars indicate s.e.m. (*n*=3). (**e**) Indel frequencies at three *AAVS1* sites targeted by CjCas9 (orange) and SaCas9 (violet).

We then determined genome-wide specificities of CjCas9 using nuclease-digested whole-genome sequencing (WGS; Digenome-seq)^20^,^21^,^22^. Cell-free genomic DNA digested with CjCas9 *in vitro* was subjected to WGS. Uniform cleavage patterns corresponding to on-target and off-target cleavage sites were computationally identified. CjCas9 designed to target 6 different DNA regions cleaved between one and 27 sites (7±4 sites, on average) in the human or mouse genome (Fig. 3a–d). In parallel, we used Digenome-seq to test three SpCas9 nucleases designed to cleave sites that overlapped with CjCas9 target sites. SpCas9 cleaved 15 to 147 sites (70±40 sites, on average) in the human or mouse genome, in line with our previous results showing that SpCas9 targeted to 11 different sites cleaved 90±30 sites in the human genome^21^. One target site in the human genome contained a 5′-NGGNACAC-3′ PAM recognized by both CjCas9 and SpCas9. CjCas9 and SpCas9 cleaved human genomic DNA at 5 sites and 45 sites, respectively, although the two Cas9 orthologues showed comparable indel frequencies at this particular site. Thus, the higher specificity of CjCas9 was not gained at the expense of its lower editing efficiency. Strikingly, two CjCas9 nucleases targeting the *Rosa26* locus or the *Vegfa* gene in the mouse genome cleaved genomic DNA only at the single on-target site, reminiscent of the remarkable specificity of Cpf1 nucleases^22^,^23^. Sequence logos obtained computationally by comparing *in vitro* cleavage sites with each other unambiguously showed that these sites contained 5′-NNNNACAC-3′ or 5′-NNNNRYAC-3′ PAMs.

We chose the most promiscuous CjCas9 nuclease, which cleaved genomic DNA at 27 sites, and performed targeted amplicon sequencing to measure indel frequencies in human cells (Supplementary Table 2). Indels occurred at the on-target site but were not detectably induced at the other *in vitro* cleavage sites. Taken together, these results show that CjCas9 nucleases are highly specific in human cells. The remarkable specificity of CjCas9 can be at least partially attributed to its extended, 22 nucleotides target and 4 nucleotides PAM sequences, compared to the 20 nucleotides target and 2 nucleotides PAM sequences recognized by SpCas9.

### Efficiency and specificity of CjCas9

We compared genome editing efficiencies and specificities of CjCas9 with those of SaCas9. We chose 3 overlapping sites in the human genome with 5′-NNGRRTAC-3′ PAM sequences, which can be targeted by both CjCas9 and SaCas9. CjCas9 and SaCas9 generated indels at these sites in HEK 293 cells with comparable frequencies of 35±7% and 36±13%, respectively (Fig. 3e). We then determined genome-wide specificities of CjCas9 and SaCas9 nucleases targeted to two of these overlapping sites using Digenome-seq (Fig. 3f). CjCas9 and SaCas9 targeted to the *AAVS1*-TS16 site cleaved human genomic DNA at 59 and 118 sites, respectively. CjCas9 and SaCas9 targeted to the *AAVS1*-TS39 site were more specific than were those targeted to the TS16 site, cleaving human genomic DNA at 2 and 15 sites, respectively. Note that CjCas9 targeted to the TS16 site was more efficient than SaCas9 in terms of on-target indel frequency (46% versus 28%), suggesting that the higher specificity of CjCas9 was not gained at the expense of a lower on-target efficiency. These results demonstrate that CjCas9 is as efficient as SaCas9 but is more specific than SaCas9 at least at the sites we tested in human cells.

### All-in-one AAV vector for *in vivo* genome editing

**Figure 4:**
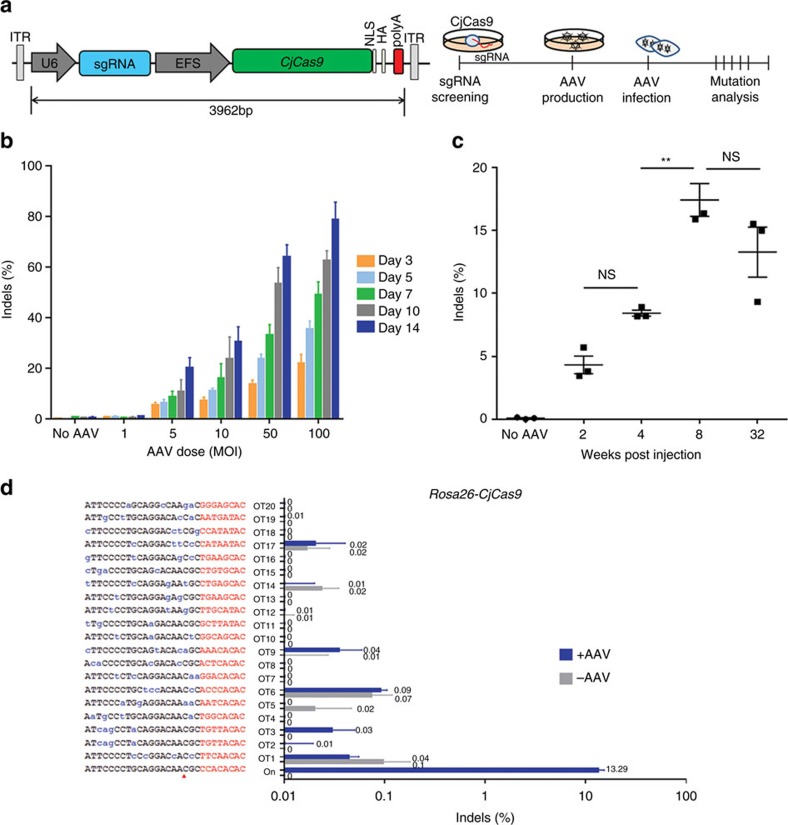
AAV-mediated mutagenesis *in vitro* and *in vivo*. (**a**) AAV vector encoding CjCas9 and its sgRNA. (**b**) Indel frequencies at the *Rosa26* target site in mouse C2C12 myotubes infected with AAV-CjCas9. (**c**) Indel frequencies at the *Rosa26* target site in TA muscles of C57BL6J mice injected with AAV-CjCas9 measured at 2, 4, 8 and 32 weeks after injection. One-way ANOVA and Tukey's *post hoc* tests, **P<0.01, NS, not significant. See also Supplementary Fig. 3. (**d**) No off-target indels were detected at 20 homologous sites that differed from the on-target site by up to 4 nucleotides in the mouse genome. Genomic DNA isolated from AAV-CjCas9-injected TA muscles of C57BL6J mice mice at 32 weeks after injection was analysed by targeted deep sequencing. Mismatched nucleotides are shown in blue and PAM sequences in red. Red arrows indicate cleavage positions within the 20-bp target sequences. Error bars indicate s.e.m. (*n*=3).

With its small size, the CjCas9 gene plus a sgRNA sequence can be packaged into an all-in-one AAV vector (Fig. 4a). CjCas9 directed to the *Rosa26* locus was expressed in C2C12 mouse myotubes using an AAV serotype DJ (AAVDJ) vector. Indels accumulated at the target site in a time- and dose-dependent manner with a frequency of up to 79±7% (Fig. 4b). Because no sites other than the on-target site were cleaved by this particular CjCas9 nuclease in the mouse genome, as shown above using Digenome-seq, we identified, using Cas-OFFinder^24^, potential off-target sites that differed from the on-target site by up to 4 nucleotides in the genome. No indels were detected at the resulting 20 homologous sites by targeted deep sequencing even at day 14 post-infection (Supplementary Fig. 3; Supplementary Table 3), confirming the high specificity of this CjCas9 nuclease.

We next packaged the muscle-specific Spc512 promoter-driven CjCas9 and the U6 promoter-driven *Rosa26*-specific sgRNA into a muscle-tropic AAV serotype 9 (AAV9) vector^25^ (Fig. 4c). The resulting virus was administered via intramuscular injection into tibialis anterior (TA) muscles of C57BL6J mice (*n*=3 per group). CjCas9-induced indels were observed at the target site in TA muscles with a frequency of 17±1% and 13±2%, 8 weeks and 32 weeks, respectively, after injection. No indels were detectably induced at the 20 possible off-target sites in TA muscles even 32 weeks after injection (Fig. 4d and Supplementary Table 3), although CjCas9 was still expressed (Supplementary Fig. 4). This result suggests that a long-term expression of CjCas9 via AAV *in vivo* does not necessarily aggravate off-target effects.

### *In vivo* genome editing in the mouse retina

**Figure 5:**
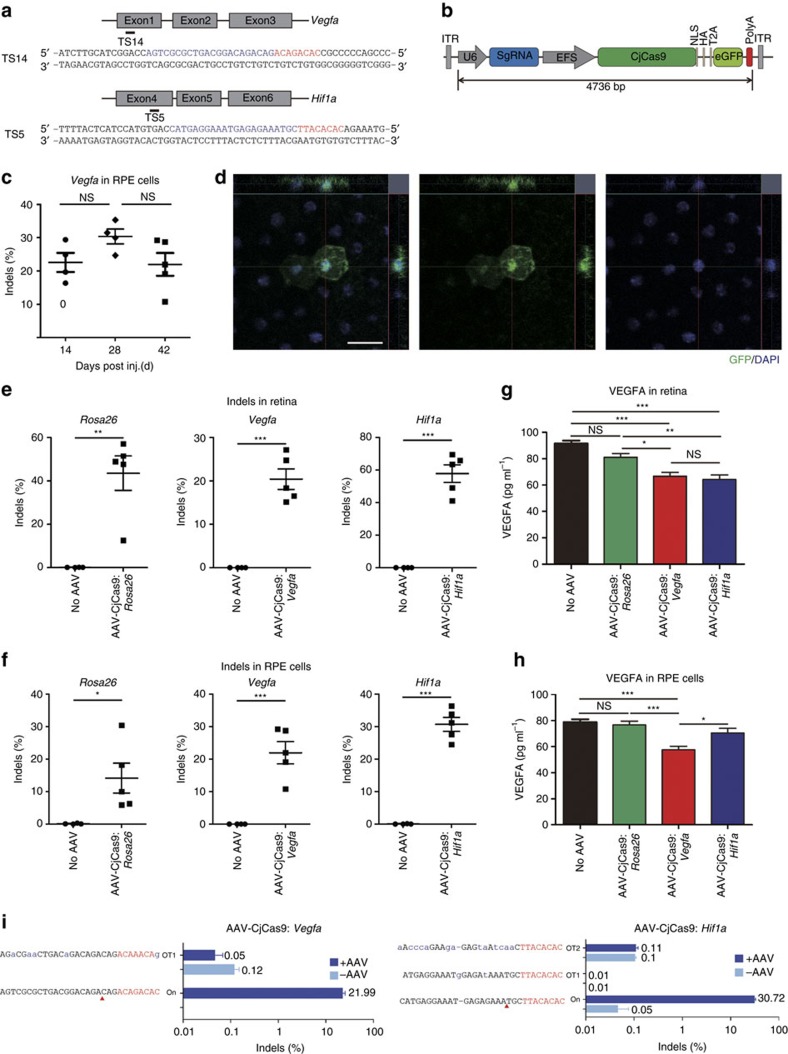
*In vivo* genome editing with CjCas9 in the retina and retinal pigment epithelium. (**a**) The CjCas9 target sequences in *Vegfa* and *Hif1a/HIF1A* genes. The PAM sequence and the sgRNA target sequence are shown in red and blue, respectively. (**b**) All-in-one AAV vector encoding CjCas9. (**c**) Indel frequencies at the *Vegfa* target site were analysed in RPE cells using deep sequencing at day 14, 28 and 42 post-intravitreal injection of AAV-CjCas9: *Vegfa*. Error bars indicate s.e.m. (*n*=4–5). One-way ANOVA and Tukey's *post hoc* tests, NS, not significant. (**d**) Representative confocal images of *in vivo* eGFP expression in RPE cells of AAV-CjCas9-injected mice 6 weeks after injection (*n*=6). eGFP was stained with anti-GFP antibody (green). Nuclei were counter-stained with DAPI (blue). Scale bar, 20 μm. (**e**–**i**) At day 42 post injection of AAV-CjCas9, indel frequencies and Vegfa protein levels were measured in retina and RPE cells using deep sequencing and ELISA, respectively. (**e**,**f**) Indel frequencies at the *Rosa26*, *Vegfa* and *Hif1a* target sites in the retina (**e**) and RPE cells (**f**). Error bars indicate s.e.m. (*n*=4 for AAV-uninjected control, *n*=5 for AAV-CjCas9). Student's *t*-tests, *P<0.05, ***P<0.001. (**g**,**h**) VEGFA levels measured by ELISA in the retina (**g**) and RPE cells (**h**), respectively. Error bars indicate s.e.m. (*n*=6–7). One-way ANOVA and Tukey's *post hoc* tests, *P<0.05, ***P<0.001. (**i**) Indel frequencies at *in vitro* cleavage sites identified by Digenome-seq. Genomic DNA isolated from RPE cells treated with AAV-CjCas9 at 6 weeks post injection was subjected to targeted deep sequencing. Mismatched nucleotides are shown in blue and PAM sequences in red. Red arrows indicate cleavage positions within the 22-bp target sequences.

To show that CjCas9 can be expressed via AAV in other tissues such as retina in mice and to investigate the therapeutic potential of CjCas9-mediated gene surgery for the treatment of age-related macular degeneration (AMD), a leading cause of blindness in adults, we prepared an AAV9 vector encoding CjCas9 under the control of the elongation factor-1 short (EFS) promoter, enhanced green fluorescent protein (eGFP) linked to the C terminus of CjCas9 with the self-cleaving T2A peptide, and a U6 promoter-driven sgRNA specific to the *Vegfa* or *Hif1a* gene, whose expression in the retina is associated with choroidal neovascularization (CNV)^26^,^27^ (Fig. 5a,b). We monitored the expression of CjCas9 in the eye and measured indel frequencies using targeted deep sequencing and VEGFA protein levels using ELISA, 6 weeks after the resulting viruses were administered into the eye via intravitreal injection.

In retinal pigment epithelium (RPE) cells, primary target cells for the treatment of AMD, AAV encoding the *Vegfa*-specific CjCas9 (AAV-CjCas9: *Vegfa*) achieved indels with frequencies that ranged from 22 to 30% at day 14, 28, and 42 post injection (Fig. 5c). As expected, CjCas9-linked eGFP was expressed in RPE cells (Fig. 5d and Supplementary Fig. 5). At day 42 post injection, CjCas9-induced indels were observed at *Rosa26*, *Vegfa*, and *Hif1a* target sites in the retina with a frequency of 44±18%, 20±5, 58±12, respectively (Fig. 5e) and in RPE cells with a frequency of 14±5%, 22±3%, and 31±2%, respectively (Fig. 5f). As expected, the VEGFA protein level measured using ELISA was decreased in the retina treated with AAV encoding the *Vegfa*- or *Hif1a*-specific CjCas9 (AAV-CjCas9: *Vegfa* and *Hif1a*) but not in those treated with AAV-CjCas9: *Rosa26* (Fig. 5g). The VEGFA protein level was also decreased in RPE cells treated with the AAV-CjCas9: *Vegfa*. However, the protein level was not decreased in those with the AAV-CjCas9: *Hif1a* or *Rosa26* (Fig. 5h), suggesting that VEGFA expression is differentially regulated in the retina and in RPE cells. We did not measure HIF-1α protein levels because HIF-1α is degraded under normoxia conditions. No off-target indels were detectably induced in these cells at one or two *in vitro* cleavage sites captured by Digenome-seq using the *Vegfa*-specific or the *Hif1a* specific sgRNA, respectively (Fig. 5i), ruling out the possibility that the partial suppression of VEGFA expression in the retina and RPE cells were caused by CjCas9 off-target effects.

### Therapeutic genome editing for the treatment of CNV

**Figure 6:**
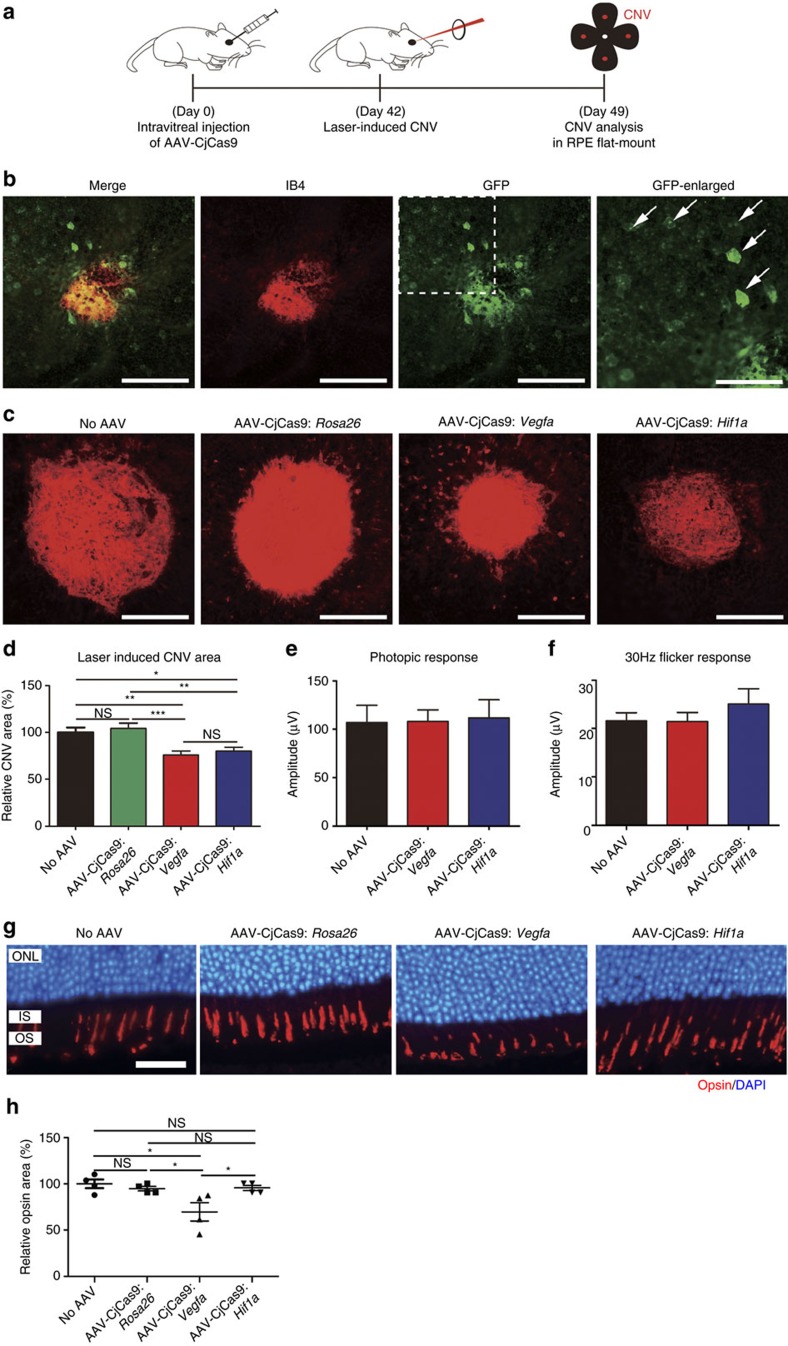
CjCas9 targeted to *Vegfa* or *Hif1a* reduces the area of laser-induced CNV in mice. (**a**) At day 42 post injection of AAV-CjCas9, mice was treated with laser to induce choroidal neovascularization (CNV). One week after laser treatment, the CNV area was analysed. (**b**) *In vivo* expression of eGFP coexpressed with CjCas9 in laser-induced CNV. Representative confocal images of eGFP expression in the RPE of laser-induced CNV, 6 weeks after injection of AAV-CjCas9: *Hif1a*. eGFP was stained with anti-GFP antibody (green), CNV was stained with anti-IB4 antibody (red), and nuclei were counter-stained with DAPI (blue). Scale bar, 200 μm. Arrows indicate eGFP-expressing RPE cells. Scale bar, 100 μm (enlarged image). (**c**) Representative laser-induced CNV stained with isolectin B4 in the mouse eye injected with AAV-CjCas9 targeted to *Rosa26*, *Vegfa* or *Hif1a*. Scale bar, 200 μm. (**d**) The CNV area. Error bars indicate s.e.m. (*n*=17–18). One-way ANOVA and Tukey's *post hoc* tests, *P<0.05; **P<0.01; ***P<0.001; NS, not significant. (**e**,**f**) At day 56 post AAV injection, full-field electroretinogram (ERG) was performed to evaluate cone function in mice. There was no significant decrease of b-wave of photopic response (**e**) and 30 Hz flicker response (**f**) in both AAV-CjCas9: *Vegfa* (*n*=8) or AAV-CjCas9: *Hif1a* (*n*=6) treated mice, compared to normal control mice (*n*=8). Error bars indicate s.e.m. (*n*=6–8). (**g**,**h**) Opsin-positive areas in the retina at day 42 post injection. (**g**) Representative images of opsin-positive areas in contact with RPE cells expressing HA-tagged CjCas9 in AAV-CjCas9: *Rosa26*, *Vegfa* or *Hif1a* injected mice compared with the AAV-uninjected negative control mice (no AAV). Opsin (red) and DAPI (blue). Scale bar, 20 μm. ONL, outer nuclear layer; IS, inner segment of photoreceptor cells; OS, outer segment of photoreceptor cells. See also Supplementary Fig. 6. (**h**) Relative opsin areas of the AAV-CjCas9-injected mice were normalized to that of the AAV-uninjected negative control mice. Error bars indicate s.e.m. (*n*=4). One-way ANOVA and Tukey's *post hoc* tests, *P<0.05.

Next, we induced CNV in the eye by laser treatment 6 weeks after injection of AAV and measured the area of CNV 1 week later (Fig. 6a). Both AAV-CjCas9: *Vegfa* and AAV-CjCas9: *Hif1a* reduced the area of CNV by 24±4% and 20±4%, respectively, compared to the AAV-uninjected negative control (Fig. 6b–d). The *Rosa26*-specific CjCas9, used as another negative control, did not show any therapeutic effect. We noted that CjCas9 linked eGFP was expressed in RPE cells surrounding CNV, which are a major source of VEGFA in laser-induced CNV (Fig. 6b). This result shows that the reduction of CNV area coincides with targeted *Vegfa* mutagenesis and partial suppression of VEGFA expression in RPE cells (Fig. 5h).

We then investigated whether the partial gene knockout of *Vegfa* and *Hif1a* in RPE cells using AAV caused any side effect. It was reported that a conditional knockout of the *Vegfa* gene but not that of the *Hif1a* gene in mouse RPE cells leads to cone dysfunction^28^. We measured cone function using full-field electroretinography (ERG) in laser-untreated CNV-free mice 8 weeks after injection of AAV-CjCas9: *Vegfa* and AAV-CjCas9: *Hif1a* (Fig. 6e,f). No significant decrease in photopic response (Fig. 6e) or 30 Hz flicker response (Fig. 6f) was observed in these mice, compared to AAV-uninjected control mice. We also measured the size of the opsin-positive area, which is closely related to cone function, in contact with RPE cells expressing CjCas9. The AAV-CjCas9: *Vegfa* reduced the size by 30±10%, compared with the AAV-uninjected control (Fig. 6g,h; Supplementary Fig. 6), suggesting that the partial *Vegfa* gene knockout using AAV can still cause local opsin dysfunction near *Vegfa*-edited RPE cells. As expected, however, the AAV-CjCas9: *Rosa26* or AAV-CjCas9: *Hif1a* did not cause any such cone dysfunction. Taken together, these results raise a concern about targeted inactivation of *Vegfa* in RPE cells using AAV and suggest that *Hif1*a could be inactivated without causing cone dysfunction to avoid neovascularization for the treatment of AMD.

## Discussion

In this report, we have presented a small and highly specific Cas9 orthologue, derived from *C. jejuni*, that can be packaged with a reporter gene in an AAV vector for efficient gene surgery *in vivo*. The small size of CjCas9, compared with other orthologues including *Neisseria meningitidis* Cas9 (ref. 29), *Streptococcus thermophilus* Cas9 (ref. 29), SpCas9 (ref. 30) and SaCas9 (refs 17, 31; Fig. 1a), allows more room for additional effectors or homology arms required for homologous recombination in AAV and other animal or plant viral vectors. Because CjCas9 is highly specific *in vitro* and *in vivo*, we expect that it will be widely used for precision genome editing in research and gene surgery in medicine.

In this study, we delivered the CjCas9 gene, its sgRNA sequence, and the GFP-coding gene to mutate three genes in two different tissues, TA muscles and eyes, in mice. CjCas9-induced indels at high frequencies in the *Vegfa* and *Hif1a* genes *in vivo*. HIF-1α is a hypoxia-inducible transcription factor that activates the transcription of *VEGF A* (ref. 32). Unlike VEGF A, a secretory protein and a primary therapeutic target for the treatment of AMD, HIF-1α has not been considered as a drug target: Indeed, HIF1α in particular and transcription factors in general cannot be targeted directly by antibodies or aptamers or small molecules. In this study, we showed that CjCas9 targeted to the *Hif1a* gene in mouse eyes inactivated the gene in RPE cells efficiently and reduced the area of CNV in a mouse model of AMD. Because the CjCas9 target site in the mouse *Hif1a* gene is perfectly conserved in the human *HIF1A* gene, the AAV presented in this study or its variants could be used for the treatment of human patients in the future. We expect that CjCas9 can be directed to other traditionally ‘undruggable' genes or non-coding sequences to broaden the range of therapeutic targets, making the entire human genome potentially druggable.

## Methods

### Animals

The care, use, and treatment of all animals in this study were in strict agreement with the ARVO statement for the Use of Animals in Ophthalmic and Vision Research and College of Veterinary Medicine and the guidelines established by the Seoul National University Institutional Animal Care and Use Committee, which granted permission to perform animal experiments. Eight-week-old, male, specific pathogen-free C57BL/6J mice (*n*=3–9) were used in this study. Mice were maintained under a 12 h dark–light cycle.

### Laser-induced CNV model

After mice were anaesthetized, pupils were dilated with an eye drop containing phenylephrine (0.5%) and tropicamide (0.5%). Laser photocoagulation was performed using an indirect head set delivery system (Iridex) and laser system (Ilooda). Laser parameters were 810 nm wave length, 200 μm spot size, 800 mW power and 70 ms exposure time. Laser burn was induced three to four times around the optic disc. Only burns that produced a bubble without vitreous haemorrhage were included in the study. Seven days later, the eyes were fixed in 4% paraformaldehyde for 1 h at room temperature. RPE complexes (RPE/choroid/sclera) were prepared for immunostaining and then incubated with isolectin-B4 (Thermo Fisher Scientific, cat. no. I21413, 1:100) and anti-GFP antibody (Abcam, ab6556, 1:100) overnight at 4 °C. The RPE complex was flat-mounted and viewed with a fluorescent microscope (Eclipse 90i, Nikon) or a confocal microscope (LSM 710, Carl Zeiss) at a magnification of × 100. The CNV area was measured using Image J software (1.47v, NIH) by blinded observers. An average of 3–4 CNV areas per eye were analysed. Each group consisted of 17–18 eyes.

### Construction of cjCas9 and sgRNA plasmids

A human codon-optimized CjCas9-coding sequence, derived from *Campylobacter jejuni* subsp. *Jejuni* NCTC 11168, was synthesized with a nuclear localization signal and an HA epitope at its C-terminal end (GeneArt Gene Synthesis, Thermo Fisher Scientific) and cloned into the p3s plasmid^4^. The trans-activating crRNA (tracrRNA) sequence and the precursor CRISPR RNA (pre-crRNA) sequence were fused with a GAAA or TGAA linker to form a sgRNA sequence (Supplementary Fig. 1). sgRNAs were transcribed under the control of the U6 promoter. We used addgene plasmid (# 61591) for SaCas9 expression.

### *In vitro* PAM identification

The recombinant Cas9 protein was expressed in *E. coli* and purified as described previously^3^. sgRNAs were transcribed using T7 RNA polymerase as described^3^. To make a randomized PAM (N_10_) library, the DNA sequence including the *AAVS1*-TS1 target site cloned in a plasmid was amplified with a randomized primer. After gel purification, the amplicon library (1 μg) was digested with the SpCas9 or CjCas9 protein and *in vitro* transcribed sgRNA for 30 min at 37 °C. Digested library was purified by column filtration and subjected to deep sequencing using Miseq (Illumina). Miseq reads that perfectly matched the reference sequence were sorted. The randomized PAM region was extracted and analysed with WebLogo.

### PAM characterization using cell-based reporter assays

The *AAVS1*-TS1 target sites with variable PAM sequences, which were randomized at position X (5′-NNNNXCAC-3′, 5′-NNNNAXAC-3′, 5′-NNNNACXC-3′ and 5′-NNNNACAX-3′), were synthesized (Macrogen, Inc.) and cloned in a surrogate reporter plasmid encoding RFP and GFP^19^. To determine optimal PAM sequences, each of the resulting reporter plasmids (100 ng) and plasmids encoding CjCas9 and its sgRNA (225 and 675 ng, respectively) were co-transfected into HEK293 cells (1 × 10^5^) using lipofectamine 2000 (Invitrogen). At day 2 post-transfection, the fraction of GFP and RFP double-positive cells was determined by flow cytometry (BD Accuri C6, BD).

### Cell culture and mutation analysis

HEK 293 (ATCC, CRL-1573) cells and mouse NIH 3T3 (ATCC, CRL-1658) cells were maintained in DMEM supplemented with 100 units per ml penicillin, 100 mg ml^−1^ streptomycin, and 10% fetal bovine serum (FBS). sgRNA plasmid (750 ng) and CjCas9 plasmid (250 ng) were transfected into cells (0.5∼1 × 10^5^) with lipofectamine 2000 (Invitrogen). After 48 h of transfection, genomic DNA was isolated using a DNeasy Blood & Tissue kit (Qiagen) and on-target or off-target loci were amplified using specific primers (Supplementary Table 4) for targeted deep sequencing. Deep-sequencing libraries were generated by PCR. TruSeq HT Dual Index primers were used to label each sample. Pooled libraries were subjected to paired-end sequencing (LAS, Inc.). Indel frequencies were calculated as described previously^20^.

### Digenome sequencing

Digenome-seq was performed as described previously^20^,^21^. Genomic DNA was isolated using a DNeasy Tissue kit (Qiagen) according to the manufacturer's instructions. Genomic DNA (8 μg) with CjCas9 or SaCas9 protein (300 nM) and sgRNA (900 nM) in a 400 μl reaction volume (100 mM NaCl, 50 mM Tris-HCl, 10 mM MgCl_2_, and 100 μg ml^−1^ BSA) and incubated the mixture for 8 h at 37 °C. Digested genomic DNA was incubated with RNase A (50 μg ml^−1^) for 30 min at 37 °C and purified again with a DNeasy Tissue kit (Qiagen). Digested DNA was fragmented using the Covaris system and ligated with adaptors for library formation. DNA libraries were subjected to WGS using an Illumina HiSeq X Ten Sequencer at Macrogen. We used the Isaac aligner to generate a Bam file using the following parameters: ver. 01.14.03.12; Human genome reference, hg19 from UCSC (original GRCh37 from NCBI, Feb. 2009), Mouse genome reference, mm10 from UCSC; Base quality cutoff, 15; Keep duplicate reads, yes; Variable read length support, yes; Realign gaps, no; and Adaptor clipping, yes (adaptor: 5′-AGATCGGAAGAGC*-3′, 5′-*GCTCTTCCGATCT-3′)^33^.

### AAV vectors encoding CjCas9 and its sgRNA sequences

AAV inverted terminal repeat-based vector plasmids carrying a sgRNA sequence and the CjCas9 gene with a nuclear localization signal and an HA tag at the C terminus were constructed. sgRNA transcription was driven by the U6 promoter and CjCas9 expression was controlled by the EFS promoter in C2C12 myoblast cells or by the Spc512 promoter in TA muscles of C57BL6J mice mice. For retinal delivery, an AAV vector encoding CjCas9 under the control of the EFS promoter, enhanced green fluorescent protein (eGFP) linked to the C terminus of CjCas9 with the self-cleaving T2A peptide, and a U6 promoter-driven sgRNA specific to the *Vegfa* or *Hif1a* gene was constructed.

### Production and characterization of AAV vectors

To produce AAV vectors, they were pseudotyped in AAVDJ or AAV9 capsids. HEK293T cells were transfected with pAAV-ITR-CjCas9-sgRNA, pAAVED2/9 and helper plasmid. HEK293T cells were cultured in DMEM with 2% FBS. Recombinant pseudotyped AAV vector stocks were generated using PEI coprecipitation with PEIpro (Polyplus-transfection) and triple-transfection with plasmids at a molar ratio of 1:1:1 in HEK293T cells. After 72 h of incubation, cells were lysed and particles were purified by iodixanol (Sigma-Aldrich) step-gradient ultracentrifugation. The number of vector genomes was determined by quantitative PCR.

### AAV transduction in mouse myoblast cells

Mouse myoblast cells were infected with AAVDJ-CjCas9 at different viral doses (multiplicity of infection (MOI): 1, 5, 10, 50, and 100 determined by quantitative PCR) and maintained in DMEM with 2% FBS. At different time points, cells were collected for targeted deep sequencing. An MOI of 1 was estimated with one infectious virus particle in 100 total viral particles determined by quantitative PCR.

### Intramuscular injection of AAV

AAV was administered to 8-week-old young adult male C57BL/6J mice anaesthetized with 2–4% isoflurane. The mice were injected intramuscularly with AAV9-CjCas9 (1 × 10^11^ viral genome) in physiological saline (40 μl) using an ultra-fine insulin syringe with a 31 G needle (BD). As a negative control, C57BL/6J mice were injected with physiological saline (40 μl) only.

### Intravitreal injection of AAV

8-week-old mice were anaesthetized with an intraperitoneal injection of a mixture of tiletamine and zolazepam (1:1, 2.25 mg per kg body weight) and xylazine hydrochloride (0.7 mg per kg body weight). AAV9-CjCas9 (2 × 10^10^ viral genome in 2 μl) was intravitreally injected using a Nanofil syringe with a 33 G blunt needle (World Precision Instruments Inc.) under an operating microscope (Leica Microsystems Ltd.).

### Immunofluorescent staining and imaging of retinal tissue

For the analysis of opsin-positive area, formalin-fixed paraffin-embedded samples were prepared at day 42 post injection (*n*=4). Cross-section samples were immunostained with anti-HA antibody (Roche, 3F10, 1:1,000), anti-opsin antibody (Millipore, AB5405, 1:1,000), and Alexa Fluor 488 or 594 antibodies (Thermo Fisher Scientific, 1:500). The opsin-positive area corresponding to RPE cells expressing HA-tagged CjCas9 was measured using Image J software (1.47v, NIH) by blinded observers. For the distribution of CjCas9 and eGFP, the eyes were fixed in 4% paraformaldehyde for 1 h at room temperature. RPE complexes (RPE/choroid/sclera) were prepared for immunostaining and then incubated with anti-GFP antibody (Abcam, ab6556, 1:100) overnight at 4 °C. After stain with Alexa Fluor 488 antibodies (1:500), the RPE flat-mounts was imaged using a confocal microscope (LSM 710, Carl Zeiss). The scanning parameters were as follows: scaling (*x*=0.042 μm per pixel, *y*=0.042 μm per pixel, *z*=0.603 μm per pixel), dimensions (*x*=1,024, *y*=1,024, channels: 2, 8-bit) with objective C-Apochromat × 40 per 1.20 W Korr M27. ZEN 2 software was used to process the images.

### Genomic DNA extraction

For DNA extraction from muscle, muscle tissue was homogenized using tungsten carbide beads (3 mm; Qiagen) and a TissueLyser II (Qiagen). For extraction from RPE, after imaging of RPE flat-mounts, tissue samples were washed in PBS. RPE cells were mechanically isolated from choroid/sclera by vortexing for 30 s in lysis buffer (NucleoSpin Tissue, Macherey-Nagel), as described^34^. Genomic DNA from the remnant choroid/sclera tissues was analysed to confirm complete isolation of RPE cells. Genomic DNA was analysed by targeted deep sequencing.

### Mouse Vegfa ELISA

At day 42 post injection, whole RPE complexes were separated from neural retina tissue and frozen for further analysis. Sample tissues were lysed with Cell Lysis Buffer (120 μl) (CST #9803) and Vegfa protein levels were measured using a mouse VEGF Quantikine ELISA kit (MMV00, R&D systems) according to the manufacturer's instructions.

### ERG analysis

Mice were dark-adapted over 16 h. Mice were anaesthetized with an intraperitoneal injection of a mixture of tiletamine and zolazepam (1:1, 2.25 mg per kg body weight) and xylazine hydrochloride (0.7 mg per kg body weight). Pupils were dilated with an eye drop containing phenylephrine (0.5%) and tropicamide (0.5%). Contact lens electrodes were placed on both eyes with a drop of methylcellose. Full-field ERGs were recorded as described^35^ by using the universal testing and electrophysiologic system 2000 (UTAS E-2000, LKC Technologies, Gaithersburg, MD). The responses were recorded at a gain of 2 k using a notch filter at 60 Hz, and were bandpass filtered between 0.1 and 1,500 Hz. In the light-adapted state (photopic), with a 30 cd/m^2^ background light to desensitize the rods and isolate cones, cone responses were recorded in response to single flashed of 0 dB for photopic response, and a flicker sequence of 30 Hz, averaging 20 responses. The amplitude of the a-wave was measured from the baseline to the lowest negative-going voltage, whereas peak b-wave amplitudes were measured from the trough of the a-wave to the highest peak of the positive b-wave.

### Western blotting

The CjCas9 protein expressed in TA muscles of C57BL/6J mice at 8 months after injection of AAV was detected using western blotting. Samples containing equal amounts of protein (20 μg) were analysed; Cas9 and GAPDH were detected with an anti-HA high affinity antibody (Abcam, ab9110, 1:5,000) and an anti-GAPDH antibody (Abcam, ab9485, 1:2,500), respectively. Goat anti-rabbit IgG-HRP antibody (Abcam, ab6721, 1:5,000) was used for signal detection. ImageQuant LAS4000 (GE healthcare) was used for digital imaging. Uncropped scans of blots in main figures are presented in Supplementary Fig. 4.

### Statistical analysis

No statistical methods were used to predetermine sample size for *in vitro* or *in vivo* experiments. All group results are expressed as mean±s.e.m., if not stated otherwise. Comparisons between groups were made using the two-tailed Student's *t*-test or one-way analysis of variance (ANOVA) and Tukey's *post hoc* tests for multiple groups. Statistical significance as compared with untreated controls was denoted with *P<0.05, **P<0.01, ***P<0.001 in the figures and figure legends. Statistical analysis was performed in Graph Pad PRISM 5.

### Data availability

The deep-sequencing data from this study have been submitted to the NCBI Sequence Read Archive under accession number SRP095501 and SRP095507. The data that support the findings of this study are available from the corresponding author upon reasonable request.

## Additional information

**How to cite this article:** Kim, E. *et al*. *In vivo* genome editing with a small Cas9 orthologue derived from *Campylobacter jejuni*. *Nat. Commun.* **8,** 14500 doi: 10.1038/ncomms14500 (2017).

**Publisher's note:** Springer Nature remains neutral with regard to jurisdictional claims in published maps and institutional affiliations.

## Supplementary Material

Supplementary Information Supplementary Figures and Supplementary Tables
