# Supplementary material for: In vivo genome editing with a small Cas9 orthologue derived from Campylobacter jejuni
Source: Nat Commun. 2017 Feb 21;8:14500. doi: 10.1038/ncomms14500 (PMC5473640; doi:10.1038/ncomms14500)
Supplement: Supplementary Information — Supplementary Figures and Supplementary Tables [file ncomms14500-s1.pdf]

Protein coding sequence of CjCas9	Size
MARILAFDIGISSIGWAFSENDELKDCGVRIFTKVENPKTGESLALPRRLARSAR<br>KRLARRKARLNHLKHLIANEFKLNYEDYQSFDESLAKAYKGSLISPYELRFRALN<br>ELLSKQDFARVILHIAKRRGYDDIKNSDDKEKGAILKAIKQNEEKLANYQSVGEY<br>LYKEYFQKFKENSKEFTNVRNKKESYERCIAQSFLKDELKLIFKKQREFGFSFSK<br>KFEEEVLSVAFYKRALKDFSHLVGNCSFFTDEKRAPKNSPLAFMFVALTRIINLL<br>NNLKNTEGILYTKDDLNALLNEVLKNGTLTYKQTKKLLGLSDDYEFKGEKGTYFI<br>EFKKYKEFIKALGEHNLSQDDLNEIAKDITLIKDEIKLKKALAKYDLNQNQIDSL<br>SKLEFKDHLNISFKALKLVTPLMLEGKKYDEACNELNLKVAINEDKKDFLPAFNE<br>TYYKDEVTNPVVLRAIKEYRKVLNALLKKYGKVHKINIELAREVGKNHSQRAKIE<br>KEQNENYKAKKDAELECEKLGLKINSKNILKLRLFKEQKEFCAYSGEKIKISDLQ<br>DEKMLEIDHIYPYSRSFDDSYMNKVLVFTKQNQEKLNQTPFEAFGNDSAKWQKIE<br>VLAKNLPTKKQKRILDKNYKDKEQKNFKDRNLNDTRYIARLVLNYTKDYLDFLPL<br>SDDENTKLNDTQKGSKVHVEAKSGMLTSALRHTWGFSAKDRNNHLHHAIDAVIIA<br>YANNSIVKAFSDFKKEQESNSAELYAKKISELDYKNKRKFFEPFSGFRQKVLDKI<br>DEIFVSKPERKKPSGALHEETFRKEEEFYQSYGGKEGVLKALELGKIRKVNGKIV<br>KNGDMFRVDIFKHKKTNKFYAVPIYTMDFALKVLPNKAVARSKKGEIKDWILMDE<br>NYEFCFSLYKDSLILIQTKDMQEPEFVYYNAFTSSTVSLIVSKHDNKFETLSKNQ<br>KILFKNANEKEVIAKSIGIQNLKVFEKYIVSALGEVTKAEFRQREDFKKSGP<u>PKK</u><br><u>KRKV</u>**YPYDVPDYA**-	1003 aa

sgRNA sequence of CjCas9	Linker
NNNNNNNNNNNNNNNNNNNNGTTTTAGTCCCTGAAAAGGGACTAAAATAAAGAGTTTGCG<br>GGACTCTGCGGGGTTACAATCCCCTAAAACCGCTTTTTT	GAAA
NNNNNNNNNNNNNNNNNNNNGTTTTAGTCCCTGAAGGGACTAAAATAAAGAGTTTGCGGG<br>ACTCTGCGGGGTTACAATCCCCTAAAACCGCTTTTTT	TGAA

Native dual guide RNA sequences of CjCas9	
NNNNNNNNNNNNNNNNNNNNNNNGUUUUAGUCCCUUUUUAAAUUUCUUUAUGGUAAAU	crRNA
AAGAAAUUUAAAAAGGGACUAAAAUAAAGAGUUUGCGGGACUCUGCGGGGUUACAAUCCC<br>CUAAAACCGCUUUU	tracrRNA

Supplementary Figure 1. CjCas9 and sgRNA structure. The amino acid sequence of the CjCas9 protein and its sgRNA sequences are shown. A nuclear localization signal (underlined) and a single HA epitope (bold) were fused to the C-terminus of the 984 aa CjCas9 protein. Two different sgRNA scaffolds were designed and tested in this study. To decrease the packaging size of the AAV virus vector, sgRNA sequences with a TGAA linker was used in *in vivo* studies.

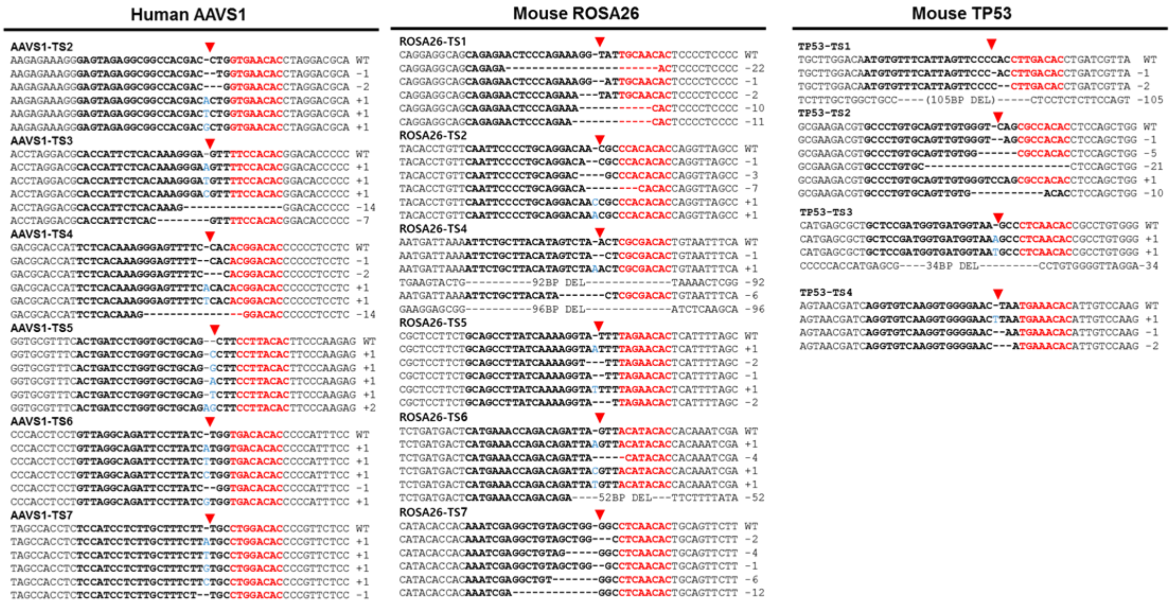

Supplementary Figure 2. CjCas9 can modify diverse sites in the human and mouse genome. Mouse *Rosa26* and *Tp53* targetable GX_{22} guide RNAs were designed and transfected into mouse NIH/3T3 cells together with CjCas9 plasmid. Genome editing efficiencies were examined by deep sequencing using genome DNA isolated from cells after 48 h of transfection. Evidence of genome editing by CjCas9 at the human *AAVS1* and mouse *Rosa26* and *Tp53* loci. Red arrows indicate cleavage positions within the 22-bp target sequences (bold), which are followed by the PAM motif (red). Inserted nucleotides are shown in light blue. The number of CjCas9-mediated inserted (+) or deleted (-) nucleotides is indicated to the right. Error bars indicate s.e.m. (n = 3).

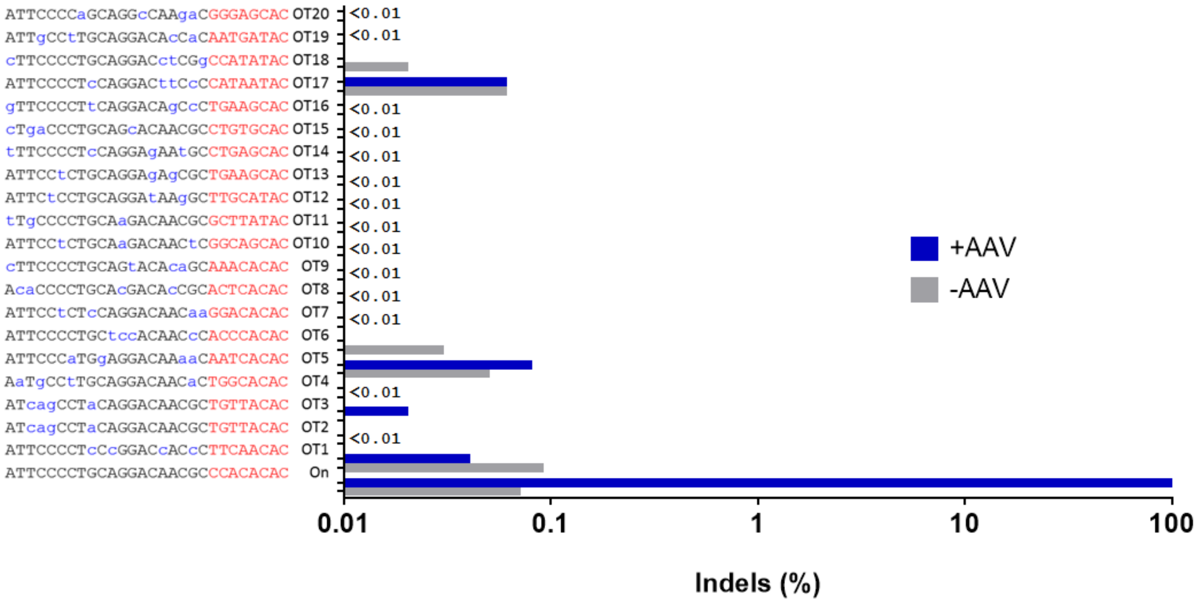

Supplementary Figure 3. Mutation frequencies at potential off-target sites measured by targeted deep sequencing.

Twenty-one possible off-target sites in the genome that differed from the on-target site by up to 4 nucleotides were examined by targeted deep sequencing in C2C12 mouse myoblast cells infected with AAVDJ-CjCas9 at day 14 post-injection. The mismatched nucleotides are shown in blue and the PAM sequences in red.

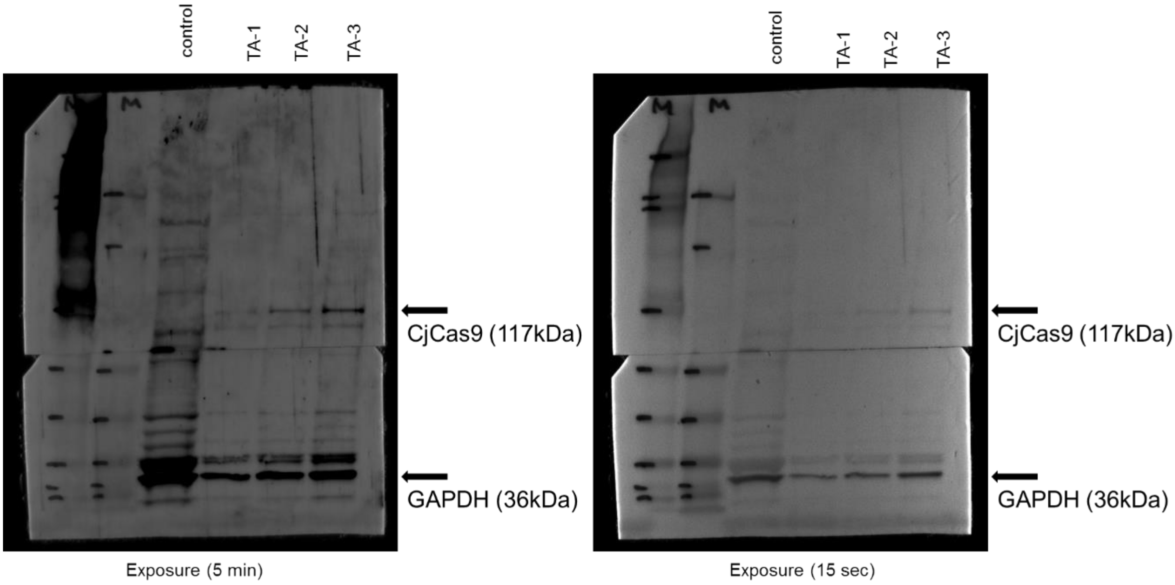

Supplementary Figure 4. Western blotting of CjCas9.

Representative western blot analysis detecting the CjCas9 protein in TA muscles of C57BL/6 mice 8 months after injection with AAV9-CjCas9 (n = 3). Uninjected TA muscles were used as the negative control. TA-1, -2, and -3 represent TA muscle samples from three C57BL/6 mice injected with AAV.

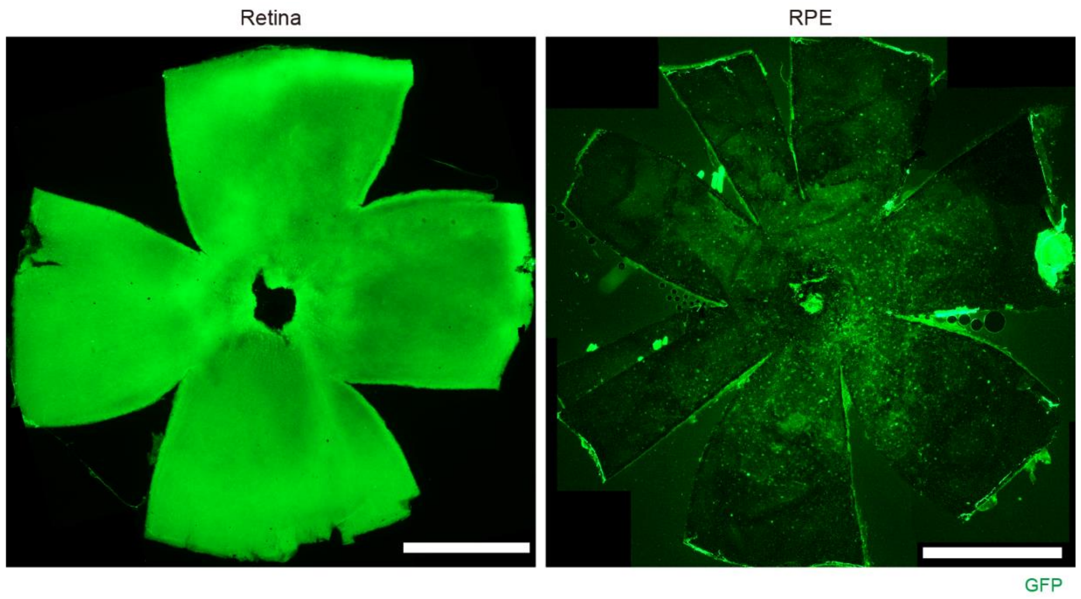

Supplementary Figure 5. Viral transduction efficiency in retina and RPE at 6 weeks post injection of AAV9-CjCas9-Hif1a.

Representative images of eGFP expression in retina and RPE cells of AAV-CjCas9-Hif1a-injected mice 6 weeks after intravitreal injection (n = 6). eGFP was stained with anti-GFP antibody (green). Scale bar is 1 mm.

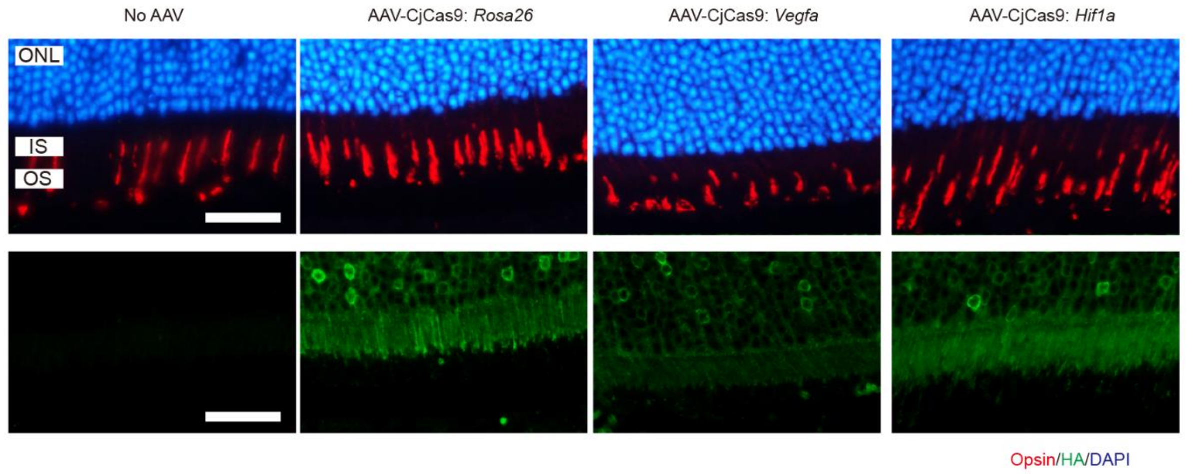

Supplementary Figure 6. Opsin positive areas in the retinas of AAV9-CjCas9-injected mice at 6 weeks post injection.

Representative images of opsin positive areas corresponding to RPE cells expressing HA-tagged CjCas9 in AAV-CjCas9: *Rosa26*, *Vegfa*, or *Hif1a* injected mice compared to the AAV-uninjected negative control mice (no AAV). Opsin (red), HA (green), and DAPI (blue). Scale bar is 20 μm. ONL: outer nuclear layer, IS: inner segment of photoreceptor cells, OS: outer segment of photoreceptor cells.

Supplementary Table 1. CjCas9 sgRNA list used in this study.

	Target site	SgRNA	Target sequences (5' to 3')	PAM (5' to 3')
1		*AAVS1*-TS1	GAATATAAGGTGGTCCCAGCTCGGGGACAC	NNNNACAC
2		*AAVS1*-TS2	GAGTAGAGGCGGCCACGACCTGGTGAACAC	NNNNACAC
3		*AAVS1*-TS3	CACCATTCTCACAAAGGGAGTTTTCCACAC	NNNNACAC
4		*AAVS1*-TS4	TCTCACAAAGGGAGTTTTCCACACGGACAC	NNNNACAC
5		*AAVS1*-TS5	ACTGATCCTGGTGCTGCAGCTTCCTTACAC	NNNNACAC
6		*AAVS1*-TS6	GTTAGGCAGATTCCTTATCTGGTGACACAC	NNNNACAC
7		*AAVS1*-TS7	TCCATCCTCTTGCTTTCTTTGCCTGGACAC	NNNNACAC
8		*AAVS1*-TS8	GGAGTGTGACAGCCTGGGGCCCAGGCACAC	NNNNACAC
9		*AAVS1*-TS9	AGGGAGCCACGAAAACAGATCCAGGGACAC	NNNNACAC
10		*AAVS1*-TS10	TCCCTCTGGAGTCCCGACTTGCTGGGACAC	NNNNACAC
11		*AAVS1*-TS11	CTGGTGTGCTGTGTGTGGGGTGGGGGACAC	NNNNACAC
12		*AAVS1*-TS12	GCCATGCGGGAGGACTAGCTAGTGGGACAC	NNNNACAC
13		*AAVS1*-TS15	CACCCCAATGCTCCAGGCCTCCTGGGATAC	NNNNATAC
14		*AAVS1*-TS16	GTGGGGTGAGGAGAAGGCTGGGAGGGATAC	NNNNATAC
15		*AAVS1*-TS17	AAGTTATGAGCAATTTCTGCAGGAAAATAC	NNNNATAC
16		*AAVS1*-TS18	GCAGGAGGTTTTCCTTGTGGCAGGAGATAC	NNNNATAC
17		*AAVS1*-TS19	AAACCAAAACAAAAAAGGCAATTTATATAC	NNNNATAC
18		*AAVS1*-TS20	TCTCAGAGTGAAAAAAATGTGGTAAAATAC	NNNNATAC
19	Human *AAVS1*	*AAVS1*-TS21	GCTCCGACCTCTTACTACTGCAAACAATAC	NNNNATAC
20		*AAVS1*-TS22	GCCACGACCTGGTGAACACCTAGGACGCAC	NNNNGCAC
21		*AAVS1*-TS23	AGCTGCAGCACCAGGATCAGTGAAACGCAC	NNNNGCAC
22		*AAVS1*-TS24	CTCTTGGGAAGTGTAAGGAAGCTGCAGCAC	NNNNGCAC
23		*AAVS1*-TS25	GGCCTTATCTCACAGGTAAAACTGACGCAC	NNNNGCAC
24		*AAVS1*-TS26	GCCATGACAGGGGGCTGGAAGAGCTAGCAC	NNNNGCAC
25		*AAVS1*-TS27	TGCTGCCCAAGGATGCTCTTTCCGGAGCAC	NNNNGCAC
26		*AAVS1*-TS28	GGCTCTAGGCATAGCTTGATCCAGGGGCAC	NNNNGCAC
27		*AAVS1*-TS29	TGGCATGGCTCTAGTGCTTTCCAGGGGCAC	NNNNGCAC
28		*AAVS1*-TS30	GCTGTTTGTGGTGGTGTTGCTGTGGGGCAC	NNNNGCAC
29		*AAVS1*-TS31	GAAGGGGGCTGCAGGGGAGGGGTGGAGCAC	NNNNGCAC
30		*AAVS1*-TS32	GCCGGTTAATGTGGCTCTGGTTCTGGGTAC	NNNNGTAC
31		*AAVS1*-TS33	CTGTGGGGTGGAGGGGACAGATAAAAGTAC	NNNNGTAC
32		*AAVS1*-TS34	GAGGTGGCTAAAGCCAGGGAGACGGGGTAC	NNNNGTAC
33		*AAVS1*-TS35	CCAAGTGGTTGATAAACCCACGTGGGGTAC	NNNNGTAC
34		*AAVS1*-TS36	CATTCTGAGACTGTGTTGCAGGCATTGTAC	NNNNGTAC
35		*AAVS1*-TS37	CTGGACCCCATATGAAGGCTCCTGGGGTAC	NNNNGTAC
36		*AAVS1*-TS38	CTGAGTGGGACACACGACTGCATGGAGTAC	NNNNGTAC
37		*AAVS1*-TS39	GATTGTGCTGTCAGGAGCTCGGGGGAGTAC	NNNNGTAC

38		*Rosa26*-TS1	CAGAGAACTCCCAGAAAGGTATTGCAACAC	NNNNACAC
39		*Rosa26*-TS2	CAATTCCCCTGCAGGACAACGCCCACACAC	NNNNACAC
40		*Rosa26*-TS3	GTGGGAAGTCTTGTCCCTCCAATTTTACAC	NNNNACAC
41	Mouse *Rosa26*	*Rosa26*-TS4	ATTCTGCTTACATAGTCTAACTCGCGACAC	NNNNACAC
42		*Rosa26*-TS5	GCAGCCTTATCAAAAGGTATTTTAGAACAC	NNNNACAC
43		*Rosa26*-TS6	CATGAAACCAGACAGATTAGTTACATACAC	NNNNACAC
44		*Rosa26*-TS7	AAATCGAGGCTGTAGCTGGGGCCTCAACAC	NNNNACAC
45		*Rosa26*-TS8	CTGCAGTTCTTTTATAACTCCTTAGTACAC	NNNNACAC
46		*Tp53*-TS1	ATGTGTTTCATTAGTTCCCCACCTTGACAC	NNNNACAC
47	Mouse *Tp53*	*Tp53*-TS2	GCCCTGTGCAGTTGTGGGTCAGCGCCACAC	NNNNACAC
48		*Tp53*-TS3	GCTCCGATGGTGATGGTAAGCCCTCAACAC	NNNNACAC
49		*Tp53*-TS4	AGGTGTCAAGGTGGGGAACTAATGAAACAC	NNNNACAC
50	Mouse *Vegfa*	*Vegfa*-TS13	AGCAGCCTGCACAGCGCATCAGCGGCACAC	NNNNACAC
51		*Vegfa*-TS14	AGTCGCGCTGACGGACAGACAGACAGACAC	NNNNACAC
52	Mouse *Hif1a*	*Hif1a*-TS5	CATGAGGAAATGAGAGAAATGCTTACACAC	NNNNACAC

Supplementary Table 2. Indel frequencies at on-target and potential off-target validated by Digenome-seq.

Target		Location		Target sequence (5' to 3')	Indel frequency (%) (-) RGEN	Indel frequency (%) (+) RGEN
AAVS1-TS1	On-target	chr19	55627221	ATATAAGGTGGTCCCAGCTCGGGGACAC	0.02	11.085
	AAVS1-TS1-02	chr22	20990738	gcATctGaTGGcCCCAGCTCTGTGACAC	0.004	0.008
	AAVS1-TS1-03	chr17	17270109	gctTcAGGTGaTCCCAGCcCAGAGGTAC	0.003	0
	AAVS1-TS1-04	chr14	70581187	caAgAAGGTGaaCCCAGCTCTCACATAC	0.013	0.024
	AAVS1-TS1-05	chr17	79782954	ccttAAGGTGaTCCCAGCTCCCCGGCAC	0.03	0.041
	AAVS1-TS1-06	chr8	80240626	tTtacAGGcaaTCCCAGCTCTGAAACAC	0.043	0.059
	AAVS1-TS1-07	chr9	129141695	taAgccaGTGGTCtCAGCTCCTCCACAC	0.014	0.022
	AAVS1-TS1-08	chr21	41295936	tTgcAAGaTGGcCCCAGCTCAGAAATAC	0.011	0.008
	AAVS1-TS1-09	chr7	11346020	tcgagAGGTtaTCCCAGCTCTAGAACAC	0.02	0.034
	AAVS1-TS1-10	chr20	40758976	ccAgcAGGTGGcCCCAGCTCCTGAGCAC	0	0
	AAVS1-TS1-11	chr4	153926891	cTgacAGGagacCCCAGCTCCAACACAC	0	0
	AAVS1-TS1-12	chr22	46402289	AgAgAgGGTGGcCCCAGCcCTACGACAC	0.006	0.003
	AAVS1-TS1-13	chrX	27472673	AcAggAGaTGaTCCCAGCTCCTGCACAC	0.279	0.318
	AAVS1-TS1-14	chr7	128481430	ATAtggtGTGGTCCCAGCTCACACACAC	0.024	0.034
	AAVS1-TS1-15	chr1	30381084	cctgActaTaGTCCCAGCTCTGAGACAC	0.041	0.027
	AAVS1-TS1-16	chr22	46426607	AgAgAgGGTGGcCCCAGCcCTACGACAC	0.003	0.006
	AAVS1-TS1-17	chr2	55333369	cTcatctGTaGTCCCAGCTCAGACGCAC	0.079	0.092
	AAVS1-TS1-18	chr8	141688584	tgcacctGTGGTCCCAGCTCCCAGCTAC	0.088	0.107
	AAVS1-TS1-19	chr1	29848565	gaAggAGGcaGcCCCAGCTCCCTCACAC	0.157	0.156
	AAVS1-TS1-20	chr18	42305670	gggggAGGaGGTCCCAGCTCTGCCACAA	0.035	0.04
	AAVS1-TS1-21	chr7	142878579	gatatgtGTGGTCCCAGCTCCTGCCCAC	0.024	0.044
	AAVS1-TS1-22	chr14	104753692	ccAtcccaTGGTCCCAGCTCTCAGGCAC	0.035	0.038
	AAVS1-TS1-23	chr14	102331176	AaAcggGGTGGTCCCAGCTCTCCCACCC	0.015	0.04
	AAVS1-TS1-24	chr4	153532801	gctaAttcTGGcCCCAGCTCTGCCACAC	0.003	0.004
	AAVS1-TS1-25	chr9	83960768	ATtagtGGTGaTCCCAGCTCTAACATAT	0.032	0.035
	AAVS1-TS1-26	chr19	32268337	cTgTAAaGTGGTaCCAGCTCAGACACAC	0.043	0.039
	AAVS1-TS1-27	chr12	9085293	AgcaccGGcaGTCCCAGCTCCTTCACAC	0.21	0.227
AAVS1-TS8	On-target	chr19	55624773	GGAGTGTGACAGCCTGGGGCCCAGGCACAC	0.046	46.623
	AAVS1-TS8-02	chr19	32022965	tGAGaGTGACAGCCTGGaGCC-AGCAACAC	0.002	0.001
	AAVS1-TS8-03	chr9	136747231	tGtcccgtACAGCCTGGGGCC-TGCCACAC	0.035	0.028
	AAVS1-TS8-04	chr1	160176618	tcgcTcTGACAGCCTGGGGCCCAGACACAC	0.036	0.030
	AAVS1-TS8-05	chr3	51397720	atgtgGaGtCAaCCTGGGGCC-TCGCACAC	0.040	0.042
Vegfa-TS14	On-target	chr17	46031879	AGTCGCGCTGACGGACAGACAGACAGACAC	0.00	21.99
	Vegfa-TS14-02	chr7	43378546	AGaCGaaCTGACaGACAGACAGACAAACAg	0.12	0.05
Hif1a-TS5	On-target	chr12	73928281	CATGAGGAAATGAGAGAAATGCTTACACAC	0.05	30.72
	Hif1a-TS5-02	chr7	76970520	ATGAGGAAATgGAGAtAAATGCTCAAACAC	0.01	0.01
	Hif1a-TS5-03	chr1	24613117	aAcccaGAAgaGAGtaAtcaaCCTGTACAC	0.10	0.11

Supplementary Table 3. Indel frequencies at on-target and potential off-target predicted by Cas-OFFinder.

Target		Location	Target sequence (5' to 3')	Indel frequency (%)*		Indel frequency (%)**	
				(-) RGEN	(+) RGEN	(-) RGEN	(+) RGEN
Rosa26 -TS2	On-target	chr6	ATTCCCCTGCAGGACAACGCCCACACAC	0.00	13.29	0.07	99.64
	Rosa26-TS2-OT1	chr3	ATTCCCCTcCcGGACcACcCTTCAACAC	0.10	0.04	0.09	0.04
	Rosa26-TS2-OT2	chr5	ATcagCCTaCAGGACAACGCTGTTACAC	0.00	0.01	0	0
	Rosa26-TS2-OT3	chr1	ATcagCCTaCAGGACAACGCTGTTACAC	0.00	0.03	0.01	0.02
	Rosa26-TS2-OT4	chr1	AaTgCCtTGCAGGACAACaCTGGCACAC	0.00	0.00	0	0
	Rosa26-TS2-OT5	chr13	ATTCCCaTGgAGGACAAaaCAATCACAC	0.02	0.00	0.05	0.08
	Rosa26-TS2-OT6	chr2	ATTCCCCTGCtccACAACcCACCCACAC	0.07	0.09	0.03	0.01
	Rosa26-TS2-OT7	chr9	ATTCCtCTcCAGGACAACaaGGACACAC	0.00	0.00	0.01	0
	Rosa26-TS2-OT8	chr11	AcaCCCCTGCAcGACAcCGCACTCACAC	0.00	0.00	0	0
	Rosa26-TS2-OT9	chr11	cTTCCCCTGCAGtACAcaGCAAACACAC	0.01	0.04	0	0
	Rosa26-TS2-OT10	chr14	ATTCCtCTGCAaGACAACtCGGCAGCAC	0.00	0.00	0	0
	Rosa26-TS2-OT11	chr15	tTgCCCCTGCAaGACAACGCGCTTATAC	0.00	0.00	0	0
	Rosa26-TS2-OT12	chr6	ATTCtCCTGCAGGAtAAgGCTTGCATAC	0.01	0.01	0.01	0.01
	Rosa26-TS2-OT13	chr6	ATTCCtCTGCAGGAgAgCGCTGAAGCAC	0.00	0.00	0	0
	Rosa26-TS2-OT14	chr11	tTTCCCCTcCAGGAgAAtGCCTGAGCAC	0.02	0.01	0	0
	Rosa26-TS2-OT15	chr17	cTgaCCCTGCAGcACAACGCCTGTGCAC	0.00	0.00	0	0
	Rosa26-TS2-OT16	chr19	gTTCCCCTtCAGGACAgCcCTGAAGCAC	0.00	0.00	0	0.01
	Rosa26-TS2-OT17	chr4	ATTCCCCTcCAGGACttCcCCATAATAC	0.02	0.02	0.06	0.06
	Rosa26-TS2-OT18	chr4	cTTCCCCTGCAGGACctCGgCCATATAC	0.00	0.00	0.02	0.01
	Rosa26-TS2-OT19	chr6	ATTgCCtTGCAGGACAcCaCAATGATAC	0.00	0.01	0	0
	Rosa26-TS2-OT20	chr9	ATTCCCCaGCAGGcCAAgaCGGGAGCAC	0.00	0.00	0	0.01

*Indel frequencies (%) in Fig.4d.
**Indel frequencies (%) in Supplementary Fig. 3.

Supplementary Table 4. Primers used in this study.

	Target site	Primer-F (5' to 3')	Primer-R (5' to 3')
	AAVS1-TS1	AGGAGGAGGCCTAAGGATGG	TGTCATGGCATCTTCCAGGG
	AAVS1-TS2	GCTCTGGGCGGAGGAATATG	TCCGTGCGTCAGTTTTACCT
	AAVS1-TS3	GCTCTGGGCGGAGGAATATG	TCCGTGCGTCAGTTTTACCT
	AAVS1-TS4	GCTCTGGGCGGAGGAATATG	TCCGTGCGTCAGTTTTACCT
	AAVS1-TS5	GGGACTAGAAAGGTGAAGAGCC	GAGGATCCTGGGAGGGAGAG
	AAVS1-TS6	ATCCTCTCTGGCTCCATCGT	CCGGTTAATGTGGCTCTGGT
	AAVS1-TS7	CCCTGGAAGATGCCATGACA	CCCTTCTTGTAGGCCTGCAT
	AAVS1-TS8	GCTATGCAGGGTGGAGGAAG	TGGACTTCGGCTTTTGTCCC
	AAVS1-TS9	TCTCATCAATGCAGGCCTGG	TTCCACTGAGCACTGAAGGC
	AAVS1-TS10	TCTCATCAATGCAGGCCTGG	TTCCACTGAGCACTGAAGGC
	AAVS1-TS11	CCCCTACACCCCCATTTCAC	GTCAACAGTGACGGGGACC
	AAVS1-TS12	CAGGGCTGATTTGACCCACT	CCGTGTGGAAGGAAAGGGAG
	AAVS1-TS15	AAAACTAGGCTGTCCTGGGC	TCTACCAGGCTGCCTTTTGG
	AAVS1-TS16	GGTCTTCGTTCCTGGCTGAT	CTTTTCCCTTGGACCCCAGG
	AAVS1-TS17	GGTCTTCGTTCCTGGCTGAT	CTTTTCCCTTGGACCCCAGG
	AAVS1-TS18	ATCAAGGTCAACACAGGGGG	TCACCATGTTGACCAGGCTG
	AAVS1-TS19	TGAATCATGTCCCACCGCAT	CTGGGAGGTGGAAGATGCAG
	AAVS1-TS20	CAGCACACCAACATGGCAC	GAGCCGAGATCTCACCACTG
On-Target	*AAVS1*-TS21	AAGTTTGCTGACCCCTGCTT	GGGAAGTGGTTGGTCAGCAT
	AAVS1-TS22	GCTCTGGGCGGAGGAATATG	TCCGTGCGTCAGTTTTACCT
	AAVS1-TS23	GGGACTAGAAAGGTGAAGAGCC	GAGGATCCTGGGAGGGAGAG
	AAVS1-TS24	GGGACTAGAAAGGTGAAGAGCC	GAGGATCCTGGGAGGGAGAG
	AAVS1-TS25	CGCACCATTCTCACAAAGGG	TGCTGCAGCTTCCTTACACT
	AAVS1-TS26	CTGAAGTGGACATAGGGGCC	AGCCACCTCTCCATCCTCTT
	AAVS1-TS27	TGGGTGAGGGAGGAGAGATG	CGCTTTCTGTCTGCAGCTTG
	AAVS1-TS28	GAGGTGCTGTGTTTTGGCTG	ATTACAGGCGTGAGTCACCG
	AAVS1-TS29	AAAGAGGAAGCTGTCTCCGC	AGTCTCAGCCAGCCACTTTC
	AAVS1-TS30	TGTGTTTGCTGCTAGAGGGC	GGTGTGTGGCCATCTGTAGG
	AAVS1-TS31	TGTGTTTGCTGCTAGAGGGC	GGTGTGTGGCCATCTGTAGG
	AAVS1-TS32	AGGAGGAGGCCTAAGGATGG	TGTCATGGCATCTTCCAGGG
	AAVS1-TS33	AGGAGGAGGCCTAAGGATGG	TGTCATGGCATCTTCCAGGG
	AAVS1-TS34	CCCTGGAAGATGCCATGACA	CCCTTCTTGTAGGCCTGCAT
	AAVS1-TS35	TCCTACTCTAGCCCCCAACC	CTCTTGATTCCCGGCCAGTT
	AAVS1-TS36	ATCAAGGTCAACACAGGGGG	TCACCATGTTGACCAGGCTG
	AAVS1-TS37	GGCAAGCCCAGGACTTTAGA	GTCTTTCTGGGAGGGGATGC
	AAVS1-TS38	GACTCACACCCCATGACCTG	GGGAAAGGCCTGTGATCTCC
	AAVS1-TS39	AAAGAGGAAGCTGTCTCCGC	AGTCTCAGCCAGCCACTTTC

	Rosa26-TS2	CGGGAGTCTTCTGGGCAGGCTTAA	CCGAGGCGGATCACAAGCAA
	Vegfa-TS14	AGGGGCAAAGTGACTGACCTGC	CTTCGGTTCCTCGCGGCTCG
	Hif1a-TS5	GTCCCCATATATGAAGAGCAC	CAATATCTGACTGAAAATCACCT
Off-target	*Rosa26*-TS2-OT1	TGGCCTCAGTGAGAGAAGAAA	ATGGAGCTGTTGCCTCATTT
	Rosa26-TS2-OT2	AGAGGCTGCACAAGAAAGGA	CAGCCTCCCAAGTTCAGAGA
	Rosa26-TS2-OT3	CTTTGGCCCTGTGAAGGTTA	CTTGAAGGGTGTTCAATTGGT
	Rosa26-TS2-OT4	ACCTTGTGAGTGGGAAGCAC	TGGAGAAAACGACTCCATGA
	Rosa26-TS2-OT5	GGTCTCAAAAGGTGGCTCAG	GTGTGAATGGGGGAGAGAGA
	Rosa26-TS2-OT6	TGCCTCCCAAGTGATAGGAT	GAGAAGAGAGGCCCTTGGTC
	Rosa26-TS2-OT7	CACAGACCGTGGTTACCTGA	ACTTCAGGCAAAGGGAAACC
	Rosa26-TS2-OT8	TGTTTATGTGCAAGCCGTGT	CCATCACAGGCACTCGTCTA
	Rosa26-TS2-OT9	TGACTGAGCAGTGTGTGCAA	AAGGGAGAAGCTGGGATCAG
	Rosa26-TS2-OT10	TTTGTGCATAAAACTTGGCTTTT	GGCCAATCAAATGACCAAAC
	Rosa26-TS2-OT11	TCATGTTCGACAACGTCACA	AACAAGGCTGCCTGTCCAT
	Rosa26-TS2-OT12	AGACCCCAGAGGAAGGACTC	GGAAGAGATCACCGAATGGA
	Rosa26-TS2-OT13	TGAACCATTTAAATTTGACAGCA	CAAAGAGAGAGCAGGTCTGGA
	Rosa26-TS2-OT14	GACGGAGGGTTCTCAGTTCA	CCAGCATCTTGTGCTCTTGA
	Rosa26-TS2-OT15	CCTGCTCTAAGGTCCAGTGC	TGGGCTGGAAAGCTGACTTA
	Rosa26-TS2-OT16	TGGCTGGTTGGTTGACCTAT	GCTTCCCCTGTGAGTTCTGA
	Rosa26-TS2-OT17	TGCATTAACTTGGGGTTCTTG	AGAGCAGTGGGACAGGAGTG
	Rosa26-TS2-OT18	TGCTGTCATCTGTGTTGCAG	GCCACAATGGGGCTATAAGT
	Rosa26-TS2-OT19	CTGGGAGAAGACTGTTCAGGA	GTGTGTTCTTTGGGGTTTGG
	Rosa26-TS2-OT20	CAGGGGATCTAACACCCTCA	CACCGGTGAAGTGAAAATTCTA
	Vegfa-TS14-OT1	CCCTTCTTCCCTCTCTCCAT	CAGCGTGCTC ACCATTCTAA
	Hif1a-TS5-OT1	TGGATCCAAGCTGAGCAATG	AGCCCAGTCC ACCATCTAAC
	Hif1a-TS5-OT2	TCACATCAAAACATCACTTCGGA	TGGGGAAGTC AGAATGCAAC
